# Supplementary figures and images for: Zinc transporter SLC39A7 relieves zinc deficiency to suppress alternative macrophage activation and impairment of phagocytosis
Source: PLoS One. 2020 Jul 9;15(7):e0235776. doi: 10.1371/journal.pone.0235776 (PMC7347223; doi:10.1371/journal.pone.0235776)

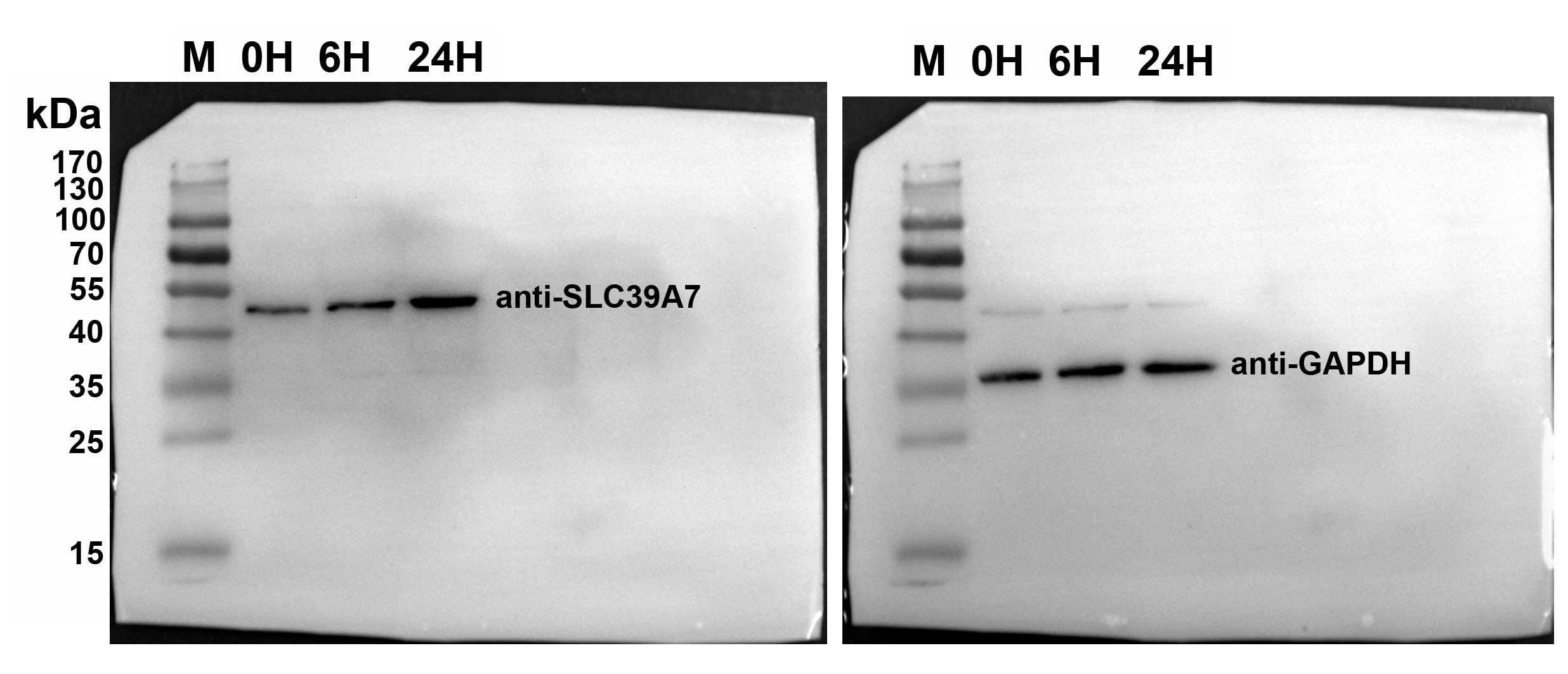

Supplement: S1 Fig — (TIF) [file pone.0235776.s001.tif]

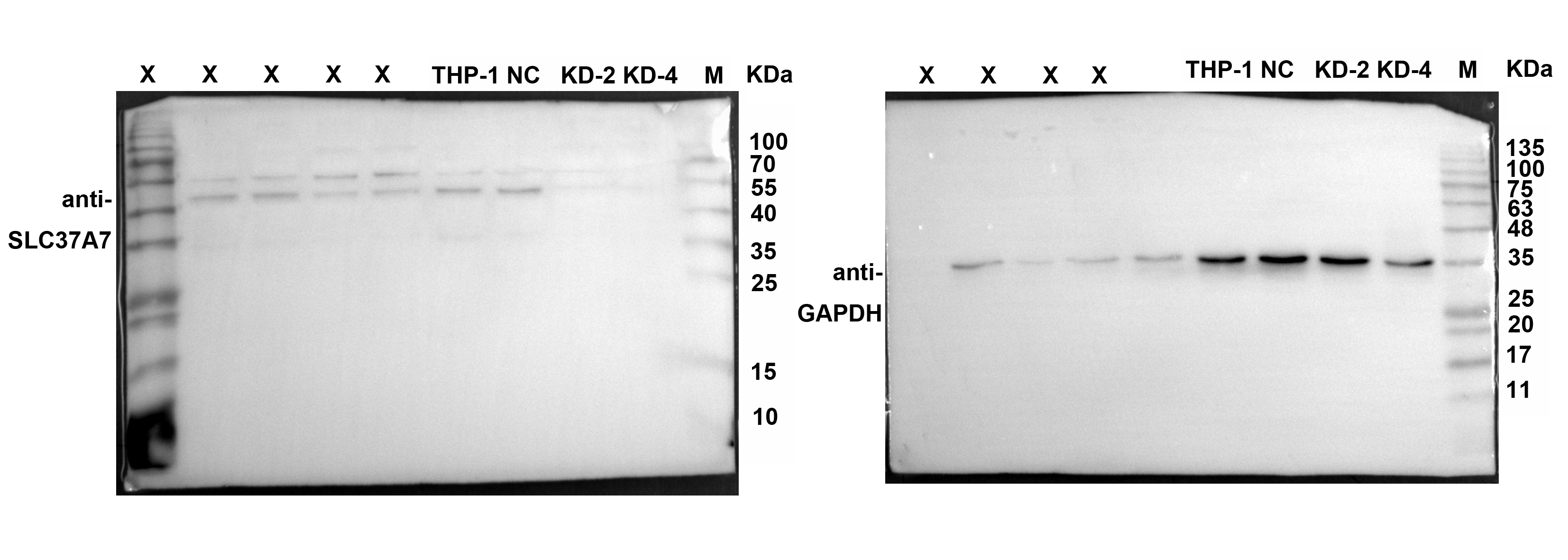

Supplement: S2 Fig — (TIF) [file pone.0235776.s002.tif]

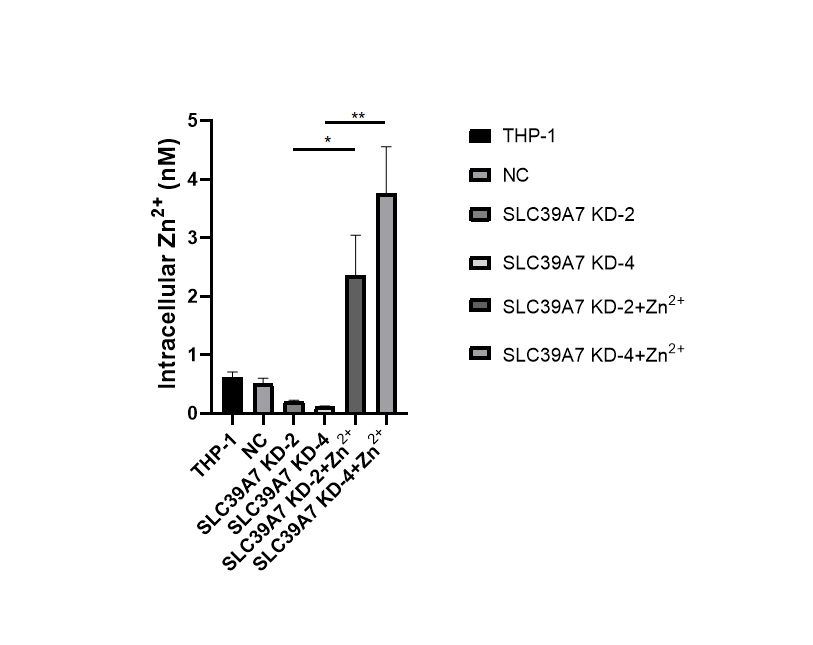

Supplement: S3 Fig — (TIF) [file pone.0235776.s003.tif]
