# Supplementary material for: Zinc transporter SLC39A7 relieves zinc deficiency to suppress alternative macrophage activation and impairment of phagocytosis
Source: PLoS One. 2020 Jul 9;15(7):e0235776. doi: 10.1371/journal.pone.0235776 (PMC7347223; doi:10.1371/journal.pone.0235776)
Supplement: S1 Table — (DOCX) [file pone.0235776.s004.docx]

**S Table 1. The list of primers**

| Name | Sequence (5’-3’) |
| --- | --- |
| SLC39A7 for | CGAAGGTGGAACGGAACTT |
| SLC39A7 rev | AGGCCTGGAAAGGATGGTAG |
| Arg-1 for | TGGACAGACTAGGAATTGGCA |
| Arg-1 rev | CCAGTCCGTCAACATCAAAACT |
| Ym1 for | GGGCTCTGCTGCAAAACTG |
| Ym1 rev | AGAGTCTCGTCATTCCACTCAG |
| Clec4e for | CTGAAACACAATGCACAGAGAGA |
| Clec4e rev | AAAGATGCGAAATGTCACAACAC |
| TLR4 for | AGTTGATCTACCAAGCCTTGAGT |
| TLR4 rev | GCTGGTTGTCCCAAAATCACTTT |
| DC-SIGN for | TCAAGCAGTATTGGAACAGAGGA |
| DC-SIGN rev | CAGGAGGCTGCGGACTTTTT |
| Dectin-1 for | GGAAGCAACACATTGGAGAATGG |
| Dectin-1 rev | AGAACCCCTGTGGTTTTGACA |
| Clec4d for | GAGCACCATGCAAAGCTCAAA |
| Clec4d rev | GCTCTCCAGTCAATAGGACAACA |
| MARCO for | CAGCGGGTAGACAACTTCACT |
| MARCO rev | TTGCTCCATCTCGTCCCATAG |
| CD11c for | CTGCAAGGGTTTACATACACGG |
| CD11c rev | GAATTTTGGCGGCATCCCTAC |
| CD206 for | CTACAAGGGATCGGGTTTATGGA |
| CD206 rev | TTGGCATTGCCTAGTAGCGTA |
| TNF-a for | CCCGAGTGACAAGCCTGTAG |
| TNF-a rev | GATGGCAGAGAGGAGGTTGAC |
| IL-6 for | ACAGCCACTCACCTCTTCAG |
| IL-6 rev | CCATCTTTTTCAGCCATCTTT |
| IL-10 for | GATGCCTTCAGCAGAGTGAA |
| IL-10 rev | GCAACCCAGGTAACCCTTAAA |
| GAPDH-For | ATCACTGCCACCCAGAAGAC |
| GAPDH-Rev | ATGAGGTCCACCACCCTGTT |
