# Supplementary material for: Zinc transporter SLC39A7 relieves zinc deficiency to suppress alternative macrophage activation and impairment of phagocytosis
Source: PLoS One. 2020 Jul 9;15(7):e0235776. doi: 10.1371/journal.pone.0235776 (PMC7347223; doi:10.1371/journal.pone.0235776)
Supplement: Attachment — Submitted filename: Rebuttal letter 20200419_KWedited2.docx [file pone.0235776.s005.docx]

**Point-to-point responses**

**1. Response to Reviewer #1**

1) ***Q:*** *manuscript is covering an interesting area some of the conclusions are not in line with the results shown and the link between asthma and BCG is not discussed. The manuscript has many English mistakes and is not fully formatted.*

**A:** We thought carefully our results and conclusion, and agreed that our conclusion was improper, so we have corrected our conclusion according our experimental results (line 43-44 and line 150-151). We agreed that we made an inaccurate conclusion: ‘These results suggested that SLC39A7 played a role in the macrophage phagocytosis through mediating the expression of cell surface protein Clec4e in sensing microbial invasion.’ This statement is not replaced with the following statement:

*Line 43-44: “Taken together, these results suggested that SLC39A7 played a role in the macrophage phagocytosis.”*

In the introduction section, we also add some information about the link between asthma and BCG (line 73-75).

*Line 73-75: “BCG is a vaccine to prevent tuberculosis and has also been used as a potent immunomodulator in the decades [17]. BCG has been reported that the protection against asthma [18].”*

We went over our manuscript again three times and corrected many mistakes and inappropriate words. The statistics and figures were done again, and the information of supplementary figures (line 89, line 94, line 133-136, line 154-156 and the supporting information section) was added in the manuscript.

2) ***Q:*** *Explain why mRNA experiments were done with MOI of 10 when all other experiments were done with MOI of 5?*

**A:** We performed the mRNA experiments at 6 h after BCG-p infection; we increased the MOI to 10 for a higher host response to the BCG-p. In the other experiments, the BCG-p at MOI of 5 was used to display the difference between control cell and knockdown cell better.

3) ***Q:*** *Figure1: No statistics on time point 24h, no indication of how many repeats were done.*

**A:** We did mistakenly maked these errors in the statistics due to our negligence. We have reproduced Fig.1A and corrected the indistinctive description using Graphpad Prism 8.0 (line 420).

4) ***Q:*** *Figure2: The decrease of proliferation is rather slow and there is no difference for the first 72h and only a small but significant increase after 96h. And yet adhesion studies omit this time point and only go until 72h.*

**A:** The data of Fig.2B was analyzed again (line 426). At the 72 h in the proliferation assay, there is difference between control and SLC39A7 knockdown cell, but it was difficult to visualize the difference, we changed the graph type of Fig.2B and showed the difference between KDs and control (NC). Moreover, because all experiments were performed at 72 h after PMA stimulation, the THP-1 cells would become macrophages that incapable of proliferation, thus the adhesion assay was done only up to 72 h. We also corrected the word of ‘three’ in the figure legend (line 425).

5) ***Q:*** *Figure3: No mentioning of sonicated vs non-sonicated anywhere in the in the text or legend (either take out the of the figures or explain in text).*

**A:** We believed the result infection of non-sonicated BCG-p in Fig.3 was irrelevant and therefore was removed in the revised verison.

6) ***Q:*** *Figure4: The authors picked the one that fits his theory best and did not discuss the unresponsiveness of KD-2. Same goes for IL-10 expression. Stats, p-values don’t match again. Stats, p-value.*

**A:** We repeated our experiments at least three times, and got same trend. We presumed that the different adhesion of the two KD cells caused the discrepancy (line 98-100, Fig. 2D). KD-4 has a bigger defect in adhesion and a more responsiveness in cytokine productions. It is interesting to note that monocytes from systemic lupus erythematosus have defects in adhesion and produce elevated levels of IL-6, TNF-a, and IL-10 than healthy monocytes (Yi et al., 2010, Arch Immunol Ther Exp, PMID 20676786). Thus, the discrepancy between KD-2 and KD-4 could reflect a differential compensatory response by the KD cells. We also measured the intracellular concentration of Zn^2+^ by Fluozin-3^TM^ AM using flow cytometry and found they were significantly decreased in the two KDs, but there was no difference between the two KDs (Figure S4). The statistics analysis was done again, and the p-value was corrected due to our careless (line 433-434).

7) ***Q:*** *Figure5: TLR4 is already up before infection and is increased upon BCG phagocytosis. Address this phenomenon. Stats, p-value*

**A:** The TLR4 signaling could be activated by extracellular inflammatory molecule HMGB1. The deficiency of SLC39A7 could induce cell death and lead to the release of HMGB1. Thus the increase of TLR4 mRNA could be induced by HMGB1 in knockdown SLC39A7 cells (line 183-186). The statistics analysis was done again, and the p-value was corrected due to our carelessness (line 449).

8) ***Q:*** *Figure6: How many repeats?*
    **A:** All experiments were done at least three times. According to reviewer’s suggestion, we measured the intracellular zinc level by Fluozin-3 AM, and added the figure in supplemental section (Figure S4, line 154-156 ), the result showed that the intracellular Zn^2+^ concentration in KDs was significantly lower than that in control cells. We also performed ELISA assays for interleukin level at 48h after BCG-p infection (Figure S3, line 133-136), the results showed that proinflammatory cytokines TNF-α and IL-6 were significantly decreased in KDs at 48 hour after BCG-p infection. The mRNA of IL-6 was inconsistent with the protein, which could be caused by the improper expression of mRNA. ER is the place of mRNA translation; the knockdown of SLC39A7 could induce the UPR response, which leads the improper translation of mRNA.

**2. Response to Reviewer #2**

1) ***Q:*** *Line 63: SLC39A7 links with asthma. The reference is missing here. Is it* [*https://www.jimmunol.org/content/197/2/655*](https://www.jimmunol.org/content/197/2/655) *? Please provide the related reference.*
    **A:** Thanks for your kindly reminder. We added the reference in the Introduction (line 71, ref.16).
2) ***Q:*** *Fig. 2: What is the difference between THP-1 cells and NC cells? However, the authors don’t stress this point and prefer to focus on the results obtained with the NC strain.*
    **A:** NC cells are THP-1 cells transfected with control plasmid containing no target sequence. Because the KD#2 and KD#4 cells are THP-1 cells transfected with plasmid containing target sequence, we thought that we should use NC cells as control cells. The phagocytosis efficiency of the THP-1 cells and NC cells are comparable, so in the other experiments we performed assays only in NC cells. We checked the proliferation rate of all cells three times and found that the proliferation rate of NC was faster than others, but we don’t know how it caused, it may be caused by the different cleaved position by Cas9.
3) ***Q:*** *it is incorrect to write ‘cell viability’ here as the authors are in fact measuring the cell number via the CCK8 assay.*
    **A:** We corrected the word of “cell viability” with cell proliferation rate (line 95, line 98).
4) ***Q:*** *Line 98 to 100: What is the timepoint of cell culture at which the SYTOX Green staining has been performed?*
    **A:** We checked the cell death with SYTOX Green staining at 72 h after PMA stimulation (line 103).
5) ***Q:*** *Legend Figure 2 C: What is the time point of culture at which the SYTOX Green staining has been performed?*
    **A:** We performed the SYTOX Green staining at 72 h after PMA stimulation, and added the timepoint in the figure 2C (line 427-430).
6) ***Q:*** *As the authors studied the transcript expression levels of genes associated with the pro-inflammatory (M1) and resolving (M2) macrophages, it would be more accurate to write that they analysed ‘the transcript expression levels of genes’ rather than ‘expression of markers’.*

**A:** We corrected the words ‘the expression of markers’ with ‘the mRNA levels of genes in all corresponsive positions (line 123, line 125, and line 126).

7) ***Q:*** *Line 133: Once again I would advised to write ‘the transcript expression levels of Clec4e was reduced…’*

**A:** We changed the word ‘expression’ with ‘the mRNA level’ (line 146).

8) ***Q:*** *Line 142-144, I don’t think the authors can come to the conclusion that ‘These results suggested that SLC39A7 played a role in the macrophage phagocytosis through mediating the expression of cell surface protein Clec4e in sensing microbial invasion’.*
    **A:** We have corrected our conclusion and deleted the words ‘mediation the expression of Clec4e in sensing microbial invasion’ (line 43-44, line 150-151).

*Line 43-44: “Taken together, these results suggested that SLC39A7 played a role in the macrophage phagocytosis.”*

*Line 150-151: “These results suggested that SLC39A7 played a role in the macrophage phagocytosis; the deficiency of SLC39A7 could reduce the transcription level of Clec4e.”*

9) ***Q:*** *Results, Paragraph 5: Why are the author not describing in the results section the differences they observed for TLR4 gene expression (Figure 5). It is one of their major finding that could also explain the differences observed with the phagocytosis?*

**A:** Thank you for your kindly suggestion, we neglected the results of TLR4 gene expression due to our focus on the Clec4e gene expression. In the new manuscript, we added the analysis of TLR4 gene expression (line 146 and line 228-231).

*Line 146: “The results showed that the mRNA level of receptors Clec4e (Mincle) was significantly decreased and TLR4 mRNA was increased in SLC39A7 knockdown cells (Fig.5).”*

*Line 183-186: “The expression of TLR4 was slightly increased in SLC39A7 knockdown cells. The phenomenon could be caused by the cell death in SLC39A7 knockdown cells. The deficiency of SLC39A7 leads to the cell death and releases the extracellular inflammatory factor HMGB1, which could activate TLR4 expression [29].”*

10) ***Q:*** *Line 179: ‘The expression of TLR4’, please add ‘gene’ as you are looking for the transcript and not the protein.*
     **A:** Thank you for your advice again, we corrected ‘the expression of TLR4’ with ‘the TLR4 mRNA level’ (line 146).
11) ***Q:*** *Line 196: ‘the pro-inflammatory cytokines TNF-a’, please add ‘the gene coding for ‘. Same thing on line 197 for IL-10 and line 200 for IL-6.*
     **A:** We corrected all ‘the expression of’ with ‘the gene expression of’ in the RT-PCR results (line 129, line 130, and line 131).
12) ***Q:*** *there is no mention of supplement figures inside the result section of the manuscript.*
     **A:** We added the supplemental figures at their corresponsive positions in the manuscript (line 89, line 94, line 136, line 156, and line 291-296).
13) ***Q:*** *there is no obvious reference about these flow cytometry experiments in the rest of the manuscript.*
     **A:** In the Figure 3B and 6A, we performed the phagocytosis assay by flow cytometry. We also added a reference (ref.42, [Kordon AO](https://www.ncbi.nlm.nih.gov/pubmed/?term=Kordon%20AO%5BAuthor%5D&cauthor=true&cauthor_uid=29375507), et al., 2017, [Front Microbiol.](https://www.ncbi.nlm.nih.gov/pubmed/29375507) doi: 10.3389/fmicb.2017.02638.) in the method of phagocytosis assay.
14) ***Q:*** *Minor comments: I think this manuscript would benefit from citing and discussing recent important works done on SLC39A7 in B cell development (doi: 10.1038/s41590-018-0295-8) and glycolysis (doi: 10.1371/journal.pone.0079316. eCollection 2013.)*

**A:** Thank you for the suggestion. We added discussion about the SLC39A7 had reported in B cell development and glycolysis, all these results hinted the importance of SLC39A7 in immune-metabolism (line 65-68 and line 214-218).

*Line 65-68 in Introduction: “Recent data shows that SLC39A7 is implicated in glucose metabolism and glycemic control in skeletal muscle cells [13, 14]. In the immune cells, it was reported that SLC39A7 has essential role in B cell development; the intact of SLC39A7 was required for BCR signaling [15].”*

*Line 214-218 in Discussion: “Recent data shows that SLC39A7 is implicated in glucose metabolism and glycemic control in skeletal muscle cells [13, 14]. SLC39A7 has essential role in B cell development, SLC39A7 was required for BCR signaling [15]. All these observations showed that SLC39A7 played an essential role in the host immune metabolism. The function of SLC39A7 should be further investigated.”*
