# Supplementary material for: Zinc transporter SLC39A7 relieves zinc deficiency to suppress alternative macrophage activation and impairment of phagocytosis
Source: PLoS One. 2020 Jul 9;15(7):e0235776. doi: 10.1371/journal.pone.0235776 (PMC7347223; doi:10.1371/journal.pone.0235776)
Supplement: Attachment — Submitted filename: rebuttal letter 20200618_SECOND REVISION.docx [file pone.0235776.s006.docx]

1)	***Q：****The main finding of the study appears to be that knockdown of SLC39A7 reduces phagocytosis of BCG, through the depletion of intracellular Zn levels, which potentially results in reduced expression of Clec4e. However, this is not clear from the text. Some sections of the text imply that SLC39A7 is directly involved in the phagocytosis of BCG (i.e. line 167-168), which I do not think the authors mean to imply.*

**A**：Thank for your kindly advice. The sentence “SLC39A7 mediated the phagocytosis to BCG-p” was now replaced with “SLC39A7 played a role in the phagocytosis of BCG by THP-1 cells (line 174)”.

2)	***Q：****Fig2D: Zn does not restore adhesion in KD2, only KD4. This was raised by Reviewer 1, and addressed in the rebuttal letter, but no mention was made in the actual manuscript. An explanation in the manuscript is required. Did you measure intracellular Zn after the addition of extracellular Zn to check that intracellular Zn levels were restored in both cases?*

**A**: We performed new experiments and found that zinc supplementation substantially increased intracellular Zn2+ levels in KD2 and KD4 (Fig. S3). This elevated intracellular zinc level by supplementation was not sufficient to restore the adhesion defect in KD2. Thus, the adhesion defect in KD2 could be independent of a SLC39A7 defect that could be restored by zinc supplementation.

The following statements were added to explain the unresponsive phenotypes in KD2 (line 207-214 in Discussion):

*“One of the KD cells KD-2 did not exhibit defects in several cytokine expressions (Fig. 4A-B), and was unresponsive zinc supplementation. We presumed that the different adhesion of the two knockdown cells caused the discrepancy. KD-4 has a bigger defect in adhesion and a more responsiveness in cytokine productions. It is interesting to note that monocytes from systemic lupus erythematosus have defects in adhesion and produce elevated levels of IL-6, TNF-a, and IL-10 than healthy monocytes [38]. Thus, the discrepancy between KD-2 and KD-4 could reflect a differential compensatory response by the KD cells.”*

Note: In the new sets of experiments only upregulation of the expression of M2 macrophage marker *CD206* in response to SLC39A7 knockdown was reproduced and this upregulation could be reversed by zinc supplementation. mRNA expression of M2 macrophage marker genes *Arg-1* and *Ym-1* was either unchanged in knockdown cells or unresponsive to zinc supplementation. It is notable that the *CD206* upregulation was not seen in KD2, consistent with its unresponsiveness. Collectively, the SLC39A7-dependent regulation of M2 macrophage marker *CD206* expression (rescued by zinc supplementation) indicated that loss of SLC39A7 skewed macrophages towards alternative M2 activation.

In this second version of revised manuscript, we have one phenotype that could not be reproduced. Expression of *IL-6* was down-regulated in both BCG-p infected KD cells in two of our recent new experiments, in contrast to the previous revision where expression of *IL-6* was up-regulated (Fig. 5A). But since this *IL-6* expression down-regulation could not be restored by zinc supplementation (Fig. 5A), this down-regulation phenotype could be unrelated to the zinc transporter function of SLC39A7. Despite this observation, the release of IL-6 proteins was dependent on the zinc transporter function of SLC39A7 (Fig. 5B), indicating that SLC39A7 likely regulates IL-6 post-translationally.

3)	***Q****：Were the other phenotypes (cytokine expression and PRR expression) restored by the supplementation of exogenous Zn?*

**A:** We also assayed the PRR (*Clec4e* and *TLR4*) mRNA expression and found that the mRNA levels of *Clec4e* and *TLR4* restored by the supplementation of Zn^2+^ (line 147-149, Fig.6). We assayed the mRNA expression of cytokines (line 135-138, Fig.4, Fig.5), and found that the mRNA levels of cytokines could not restored by the supplementation of Zn^2+^, however the level of TNF-α and IL-6 proteins in the supernatants were restored by supplementation of exogenous Zn^2+^ (line 135-139, Fig.5B). IL-10 was undetectable using ELISA in uninfected and infected THP-1 cells.

4)	***Q****：Fig5: why is there no data for the Clec4e control?*

**A:** The expression of *Clec4e* in uninfected cells was negligible in comparison with that in BCG-p-infected cells where the expression of *Clec4e* increased dramatically, making it looks like there is no data for the Clec4e control. We reformatted the figure and included the data with the conditions of supplementation of Zn^2+^ (Fig.6).

5)	***Q：****Line 167-168: We found that SLC39A7 mediated the phagocytosis of BCG by THP-1 cells. Do you really mean this?*

**A:** We replaced the text with the following sentence “SLC39A7 played a role in the phagocytosis of BCG by THP-1 cells” (line 162).

6)	***Q：****Line 209: Unregulated, should this be up-regulated?*

**A:** Indeed, “up-regulated” was now used (line 207).

7)	***Q：****Figure 6 could be combined with Fig 3*

**A:** Fig 6 is now combined with Fig 3.
